# The Auxiliary Diagnosis and Imaging Characteristics of MRI Combined with CT in Patients with Cholangiocarcinoma

**DOI:** 10.1155/2021/2790958

**Published:** 2021-07-16

**Authors:** Jing Li, Yuanlin Yu, Qing He

**Affiliations:** ^1^Department of Radiology, The First Affiliated Hospital of Fujian Medical University, Fuzhou, Fujian Province, China; ^2^Department of Radiotherapy, The First Affiliated Hospital of Fujian Medical University, Fuzhou, Fujian Province, China

## Abstract

**Background:**

Patients with cholangiocarcinoma (CCA) have poor prognosis and high mortality. Therefore, early detection and early diagnosis are extremely important to control the development of CCA. This study aims to explore the diagnostic effect in patients with CCA and imaging characteristics of MRI combined with CT.

**Methods:**

109 patients with suspected CCA underwent CT and MRI before diagnosis. The examination results were compared with the “gold standard.” ROC curve was drawn to analyze the diagnostic efficacy of MRI combined with CT for CCA patients.

**Results:**

The diagnosis rate of suspected CCA patients was 95.41%. The diagnostic coincidence rate of CT and MRI examination was 89.42% and 92.31%, respectively. The diagnostic coincidence rate of MRI combined with CT examination was 100.00%. The number of CT delayed enhancement, peripheral bile duct dilatation, and hepatic capsular depression were more than those of MRI. The number of circular enhancement cases in the CT group was less than that in the MRI group. ROC curve results showed that the sensitivity and specificity of MRI combined with CT for the diagnosis of CCA patients were higher than those of MRI or CT alone.

**Conclusion:**

MRI combined with CT has high diagnostic sensitivity and specificity and can provide imaging evidence for the clinical diagnosis and treatment of CCA.

## 1. Introduction

Cholangiocarcinoma (CCA) is a primary liver cancer that occurs in the bile ducts of the liver. The prognosis of CCA patients is poor and the mortality rate is high. Most patients cannot be cured, and a small number of patients with early CCA can be clinically cured. CCA lacks typical clinical symptoms in the early stage of onset. In the advanced stage of CCA, symptoms such as upper abdominal discomfort and hepatomegaly may appear [1]. Therefore, the clinical diagnosis and treatment of CAA is more difficult.

Previous studies have shown that CCA is a tumor that occurs from the confluence of the left and right hepatic ducts (hilar) to peripheral bile duct epithelial cells [2]. Histopathological examination is a commonly used diagnostic method for CCA patients and is regarded as the “gold standard.” However, this method has poor reproducibility and requires the professional skills of doctors. It is easy to result in poor diagnosis compliance and tolerability in CCA patients [3, 4]. Therefore, simple and effective diagnostic methods are of great significance to patients with CCA.

CT is a commonly used imaging method, which has a good effect on the display of microcalcification in the lesion. MRI is more sensitive to intratumoral hemorrhage [5]. It has been shown that MRI combined with CT can give full play to the advantages of different imaging methods in the diagnosis and identification of malignant tumors [6]. At the same time, MRI combined with CT detection can evaluate the severity of the patient's disease according to different imaging features [7]. Therefore, this study aims to explore the auxiliary diagnosis in CCA patients and imaging characteristics of MRI combined with CT.

## 2. Materials and Methods

### 2.1. Patients

This is a retrospective study. 109 suspected CCA patients in the First Affiliated Hospital of Fujian Medical University were selected from February 2017 and December 2020. They are between 31 and 79 years old, with an average age of 59.39 ± 5.65 years old. The diameter of the tumor is between 23 and 135 mm, with an average diameter of 64.29 ± 6.61 mm. The clinicopathological factors of these patients are shown in Table 1. The study was approved by the Ethics Committee of the First Affiliated Hospital of Fujian Medical University. And the informed consent forms of these patients were obtained.

### 2.2. Inclusion and Exclusion Criteria

Inclusion criteria include the following: (1) patients are diagnosed by pathological examination and in line with CCA diagnostic criteria [8]. (2) Patients can tolerate and meet CT, MRI plain scan, or enhanced indications. (3) Patients have complete baseline and follow-up data.

Exclusion criteria include the following: (1) patients with mental disorders, cognitive dysfunction, or other malignant tumors; (2) patients receiving radiotherapy, chemotherapy, and biological immunotherapy before the examination; (3) patients with autoimmune system diseases and abnormal blood coagulation function.

### 2.3. Treatment Method

All patients were diagnosed by histopathological examination (gold standard). Before the diagnosis, all patients underwent CT and MRI examinations.

### 2.4. CT Examination

The Lightspeed VCT scanner is used to examine patients. Before the examination, the pathogenesis, clinical manifestations, and examination methods of CCA were explained to patients and their families to improve the cooperation of the patients. Patients were instructed to perform routine fasting examinations and routinely take 800–1000 mL of negative contrast agent before the examination. During the examination, the patient was placed in a supine position. And the relevant parameter settings were completed according to the patient's condition. Finally, the unscanned and enhanced 5 mm layer thickness image was reconstructed as a 0.625 mm image. And the coronal and sagittal image reconstruction was completed [9, 10].

### 2.5. MRI Examination

After completing the CT examination, MRI examination is performed according to the patient's wishes. The instrument is a 3.0 T Inegenia superconducting magnetic resonance scanner (Philips, Netherlands) using a 32-channel volume phased array coil. It takes about 18–23 s to complete the liver scan and imaging. Relevant parameters were set according to the patient's condition. The obtained images are transferred to the processing software. The examination results are compared with the “gold standard.” Finally, the diagnostic coincidence rate of different imaging examinations is analyzed. And the imaging characteristics of CT and MRI are recorded [11, 12].

### 2.6. Statistical Analysis

SPSS24.0 software was used to analyze the data. The difference between the two groups was analyzed by the *χ*^2^ text. The ROC curve was used to analyze the diagnostic efficacy (sensitivity and specificity) of MRI combined with CT in CCA patients. *P* < 0.05 indicates that the difference is statistically significant.

## 3. Results

### 3.1. Comparison of the Diagnostic Coincidence Rate of MRI and CT in Patients with CCA

**Figure 1:**
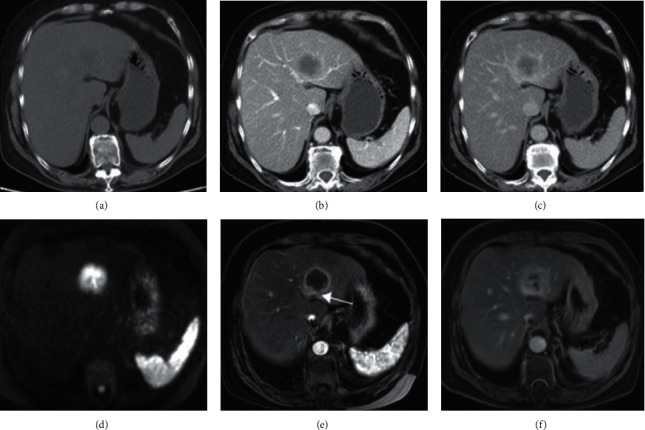
Typical pathological MRI and CT images. In 63 patients with CCA, the lesion was located in the left lobe of the liver. And all patients underwent MRI and CT examinations. (a) CT picture of a typical case, (b) a circular enhancement at the edge of the lesion in the arterial phase, (c) an enhanced inward filling in the equilibrium phase, and (d) DWI picture of the MRI shows high signal intensity. (e) A circular enhancement shows at the edge of the tumor, but the strengthening ring is incomplete. (f) The inward filling of the mass in the equilibrium phase.

Among the 109 suspected CCA patients, 104 patients were confirmed by histopathological examination. And the diagnosis rate was 95.41%. CT examination confirmed 93 cases, and the diagnosis coincidence rate was 89.42% (*P* < 0.05, Figure 1). 96 cases were confirmed by MRI examination. The coincidence rate of MRI diagnosis was 92.31% (*P* < 0.05, Figure 1). 104 cases were diagnosed by MRI combined with CT examination, and the diagnosis coincidence rate was 100.00% (*P* > 0.05, Figure 1).

### 3.2. Comparison of Imaging Characteristics between MRI and CT in Patients with CCA

The CT group and the MRI group had no significant difference in enhancement, “fast-in and fast-out” enhancement, smooth enhancement ring, flatness, hepatic lobe atrophy, DWI high signal, and abnormal perfusion (*P* > 0.05, Table 2). The number of delayed enhancement, peripheral bile duct dilation, and hepatic capsule depression in the CT group were more than those of MRI (*P* < 0.05, Table 2). The number of uneven enhancement cases in the circular-enhanced CT group was less than that of the MRI group (*P* < 0.05, Table 2).

### 3.3. Diagnostic Efficacy of MRI Combined with CT in Patients with CCA

**Figure 2:**
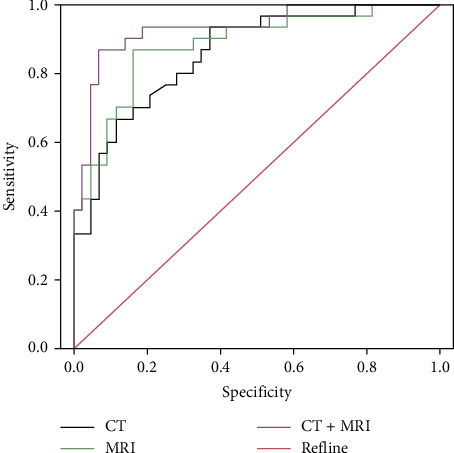
The diagnostic ROC curve of MRI combined with CT in patients with CCA.

ROC curve results showed that the sensitivity and specificity of MRI combined with CT for CCA patients were higher than those of MRI and CT alone (*P* < 0.05, Table 3, Figure 2).

## 4. Discussion

CCA is a malignant tumor with high clinical incidence. At present, it is generally believed that the incidence of CCA is related to intrahepatic bile duct stones, long-term chronic inflammation, or related physical and chemical stimulation [13, 14]. Even worse, the clinical diagnosis and treatment of CCA is difficult because of the lack of typical clinical symptoms in the early stage of CCA. Therefore, most patients are already in the middle and advanced stages at the time of diagnosis. Now, histopathological examination is still the “gold standard” for the diagnosis of patients with CCA. Although pathological examination is helpful for diagnosis, diagnostic compliance and tolerance are poor [15, 16]. Therefore, it is very important to explore new diagnostic methods of CCA in clinical practice.

In recent years, MRI combined with CT has been used in patients with CCA. Moreover, the diagnostic accuracy of MRI combined with CT is satisfactory [17]. In this study, 104 of 109 suspected CCA patients were confirmed by histopathological examination. The diagnosis rate of histopathological examination was 95.41%. In addition, the diagnosis coincidence rate of CT and MRI examination was 89.42% and 92.31%, respectively. More importantly, the diagnosis coincidence rate of CT combined with MRI reached 100.00% in this study. These results indicate that the diagnostic accuracy of MRI combined with CT is very high. Qi et al. [18] also reported that MRI combined with CT can achieve a higher detection rate in patients with CCA. Briefly, MRI combined with CT examination is helpful to the early diagnosis of CCA patients and can guide the clinical diagnosis and treatment.

It is well-known that fibrous tissue, mucin, coagulative necrosis, and malignant tumor tissue cells are the main components of the mass. And according to the area, tissue type, and distribution characteristics of the tumor, there are obvious differences between different components [19]. It has been found that the imaging manifestations of CCA mainly depend on the pathological type, the distribution of fibrous tissue, and tumor cells [20]. The tumor cells are distributed around the tumor, and the proliferating fibrous tissue is located in the center of the tumor. The contrast agent diffuses slowly in the fibrous tissue and can obtain clear images [21].

In this study, the CT group had more cases of delayed enhancement, peripheral bile duct dilation, and hepatic capsule depression than MRI. The number of uneven enhancement cases in the CT group was less than that of MRI. The above results indicate that MRI and CT have typical imaging characteristics in patients with CCA. The diagnosis of different lesions can be confirmed through imaging features. In order to further analyze the diagnostic value of MRI and CT in patients with CCA, ROC curves were drawn in this study. The results showed that the diagnostic sensitivity and specificity of MRI combined with CT in patients with CCA were higher than those of single MRI and CT. Therefore, MRI combined with CT examinations should be strengthened for patients with suspected CCA. However, the number of samples in our study is a little small. We will continue to collect data in the follow-up to better confirm our conclusions.

## 5. Conclusion

In conclusion, MRI combined with CT can achieve a high diagnostic coincidence rate in patients with CCA and has typical imaging features. Moreover, the sensitivity and specificity of the combined examination are high. Therefore, MRI combined with CT can provide imaging basis for the clinical diagnosis and treatment of CCA.

## Figures and Tables

**Table 1:** Clinicopathological information of 109 patients with cholangiocarcinoma.

Features	Gender		Symptom				Examination		
	Male	Female	Abdominal pain	Jaundice	Fever	Fatigue	CT	MRI	CT + MRI
Cases	65	44	32	16	19	29	57	29	23

**Table 2:** Comparison of imaging characteristics between MRI and CT in patients with CCA (*n* (%)).

Characteristics		CT (*n* = 57)	MRI (*n* = 29)	*χ* ^2^	*P*
Enhanced features	Annular strengthening uneven strengthening	41 (71.93)	28 (96.55)	6.351	0.035^*∗*^
	Delayed reinforcement	14 (24.56)	3 (10.34)	9.672	0.021^*∗*^
	No obvious enhancement	2 (3.51)	0 (0.00)	1.491	0.958
	“Fast-in and fast-out” enhancement	2 (3.51)	0 (0.00)	1.491	0.958
	Smooth and flat strengthening ring	0 (0.00)	0 (0.00)	0.000	1.000
	DWI high signal	57 (100.00)	29 (100.00)	0.000	1.000

Arterial passage lesions	Peripheral bile duct dilation	29 (50.88)	16 (55.17)	6.094	0.020^*∗*^
	Depressed liver capsule sign	42 (73.68)	11 (37.93)	7.103	0.013^*∗*^
	Hepatic lobe atrophy	9 (15.79)	5 (17.24)	1.593	0.615
	Abnormal perfusion	18 (31.58)	10 (34.48)	0.834	0.058

^*∗*^ *P* < 0.05.

**Table 3:** Diagnostic efficacy of MRI combined with CT in patients with CCA.

Variable	AUC	SD	*P*	95% confidence interval		Sensitivity	Specificity
				Lower limit	Upper limit		
CT	0.746	0.061	*P* ≤ 0.001	0.713	0.858	0.794	0.671
MRI	0.812	0.074	*P* ≤ 0.001	0.794	0.871	0.841	0.657
CT + MRI	0.894	0.083	*P* ≤ 0.001	0.845	0.932	0.931	0.612

## Data Availability

The datasets used and/or analyzed during the present study are available from the corresponding author on reasonable request.
